# Objective value on Apparent diffusion coefficient (ADC) map to categorize the intensity of diffusion-weighted imaging (DWI) restriction for prostate cancer detection on multiparametric prostate MRI

**DOI:** 10.1590/S1677-5538.IBJU.2018.0038

**Published:** 2018

**Authors:** Thais Caldara Mussi, Tatiana Martins, Adriano Tachibana, Pedro Nogueira Mousessian, Ronaldo Hueb Baroni

**Affiliations:** 1 Hospital Israelita Albert Einstein, São Paulo, SP, Brasil; 2 Ecoar Medicina Diagnostica, Belo Horizonte, MG, Brasil

**Keywords:** Magnetic Resonance Imaging, Prostatic Neoplasms, Prostate

## Abstract

**Purpose:**

To identify objective and subjective criteria on multiparametric prostate MRI that can be helpful for prostate cancer detection.

**Materials and Methods:**

Retrospective study, IRB approved, including 122 patients who had suspicious lesion on MRI and who underwent prostate biopsy with ultrasonography (US)/MRI imaging fusion. There were 60 patients with positive biopsies and 62 with negative biopsies. MRI of these patients were randomized and evaluated independently by two blinded radiologists. The following variables were analyzed in each lesion: morphology, contours, T2 signal, diffusion restriction (subjective impression and objective values), hyper-enhancement, contact with transition zone or prostatic contour, prostatic contour retraction, Likert and PIRADS classification.

**Results:**

Apparent diffusion coefficient (ADC) value was the best predictor of positivity for prostate cancer, with mean value of 1.08 (SD 0.20) and 1.09 mm2/sec (SD 0.24) on negative biopsies and 0.81 (SD 0.22) and 0.84 mm2/sec (SD 0.22) on positive biopsies for readers 1 and 2, respectively (p < 0.001 in both analysis). For the others categorical variables evaluated the best AUC for reader 1 was subjective intensity of diffusion restriction (AUC of 0.74) and for reader 2 was hyper-enhancement (AUC of 0.65), all inferior comparing to the value of ADC map. Interobserver agreement ranged from 0.13 to 0.75, poor in most measurements, and good or excellent (kappa > 0.6) only in lesion size and ADC values.

**Conclusions:**

Diffusion restriction with lower ADC-values is the best parameter to predict cancer on MRI prior to biopsy. Efforts to establish an ADC cutoff value would improve cancer detection, especially for less experience reader.

## INTRODUCTION

Magnetic resonance imaging (MRI) of the prostate is an important tool to detect cancer (1-3), stage the disease (4), estimate cancer aggressiveness (5, 6) and follow-up men with previous negative biopsies or on active surveillance (7, 8).

However, prostate MRI is a challenging exam, with limitations, so its description is usually performed in probability grades of having a clinically significant (CS) cancer. A Likert scale is a subjective scale firstly described by a neuroscientist and is used to grade something in levels of certainty (9). In prostate MRI the Likert scale is used to describe the probability of having prostate cancer, usually in a 5-point scale (10, 11). More recently, in an attempt to standardize the methodology and make it more objective, a multidisciplinary group published the PI-RADS (Prostate Imaging Reporting and Data System) in 2012 (12), and an updated version in 2015 (13). PI-RADS version 2 also classifies prostate lesions in a 5-point scale of probability of having a significant cancer. PI-RADS is based on subjective features of prostatic lesions, and the version 2 uses the diffusion-weighted image sequence as the dominant to categorize a lesion in the peripheral zone, and the T2-weighted imaging morphology (including heterogeneity) as the dominant sequence for lesion in transition zone. If a lesion is indeterminate, positive enhancement is used to differentiate from high probability of CS cancer in the peripheral zone and the grade of diffusion restriction is used similarly on the transition zone (12).

Nowadays, PI-RADS version 2 is widely used to standardize the communication between radiologists and urologists and also to make MRI evaluation and reports more uniform and reproducible among radiologists. It is able to improve detection, localization, characterization, and risk stratification (13). Nevertheless, some studies show low to moderate rates of interobserver agreement, even for experienced reader (14, 15).

The purpose of this study is to identify objective and subjective criteria on multiparametric prostate MRI that can help in the detection of prostate cancer and, therefore, improve MRI results.

## MATERIALS AND METHODS

### Study design

Retrospective study, institutional review board approved. We searched in our database for patients who had suspicious lesions on MRI, with prostate MRI report of high or very high probability for CS prostate cancer (PI-RADS 4 and 5) and who also underwent prostate biopsy with ultrasonography (US) / MRI imaging fusion in our institution.

Between March, 2015 and January, 2016 we found 60 patients who had PI-RADS 4 or 5 findings on MRI, including peripheral zone and transition zone lesions, with positive biopsy results for prostate cancer. Then, to eliminate a selection bias, we also included 62 patients with suspicious lesions on MRI (PI-RADS 4 and 5), but with negative biopsy results. The maximal interval between MRI and biopsies was 6 months for all patients.

If a patient had more than one suspicion lesion, the highest PI-RADS scoring lesion was counted for each patient. Images of these 122 patients were anonymized, randomized and evaluated independently by two radiologists, blinded to the clinical and histopathological results (reader 1, a board-certified radiologist with 10 years of experience in general radiology and 5 years of experience in prostate MRI, and reader 2, a board-certified radiologist with 15 years of experience in general radiology and 2 years of experience in prostate MRI). To be sure that both radiologists were reading the same lesion, a third radiologist reviewed the MRI and biopsy reports, and gave them the lesion localization (prostate lobe, prostate zone and image number on T2-weighted (T2W) imaging, matched to diffusion-weighted imaging [DWI] and dynamic contrast enhanced sequences [DCE]). The slide presentation with the imaging localization was used just to show the lesion that should be considered, however the entire exam was evaluated in a PACS (Picture Archiving and Communication System) station (KODAK / Carestream; Carestream Health, Rochester, New York, USA) independently by both radiologists. The following variables were analyzed for each lesion: morphology (wedge-shape, oval or round), contours (circumscribed, partially defined or ill-defined), T2W signal intensity (hypointense or marked hypointense), T2W signal homogeneity (homogeneous or heterogeneous), subjective diffusion restriction (mild, moderate or marked), objective value measured on apparent diffusion coefficient (ADC) map, hyper-enhancement (absent, mild, moderate or marked), contact with transition zone (absent or present) or prostatic contour (absent or present), and prostatic contour retraction (absent or present). Each lesion was also classified based on Likert and PI-RADS version 2 scales.

### MRI protocol

All patients underwent MRI on a 3-Tesla scanner: Magnetom Prisma (Siemens Medical Solutions, Erlangen, Germany) or Discovery MR 750W (GE Healthcare, Little Chalfont, United Kingdom with a phased array coil and without an endorectal coil. A routine protocol including triplanar T2W imaging using the parameters: repetition time (msec) / echo time (msec), 4700-5200 / 140-160; section thickness, 3 mm; field of view, 180 × 180; matrix 256 × 256; acceleration factor of two; and six averages. DWI were acquired with b-values of 50, 400, 800 and 1500 sec / mm2; and the ADC map was constructed based in a mono-exponential approach. DCE imaging were performed using the parameters: repetition time (msec) / echo time (msec), 3.4-3.5 / 1.4; section thickness, 3 mm; field of view, 250; matrix 224 × 224; acquiring 14 sequences with 10 seconds of temporal resolution. Extracellular gadolinium-based contrast media (Magnevist, Bayer, Leverkusen, Germany) was injected at a dose of 0.2 cc / Kg and a rate of 2 cc / sec.

### Biopsy protocol

As reference standard, the transrectal prostate biopsy with MRI / US fusion and additional samples of suspicious areas was adopted. US-guided biopsies were performed using either an Aplio 500 with Smart Fusion (Toshiba Medical System Corporation, Minato, Tokyo, Japan) or a LOGIC E9 with imaging fusion software (GE Healthcare, Little Chalfont, United Kingdom). One out of ten radiologists with at least 4 years of experience in prostate biopsy with imaging fusion MRI / US performed the biopsy. A cancer was defined as CS if Gleason score ≥ 3+4.

### Imaging evaluation

Radiologists evaluated the images using a workstation (Carestream, Rochester, New York). All sequences of the exam were interpreted in a single session and the selected parameters of that study were evaluated. ADC values were measured on the ADC map with a round ROI in the lesion in the transverse plane.

### Statistical analysis

We performed a histogram analysis and Shapiro-Wilk test to verify the distribution. Numeric variables with normal distribution were described as mean and standard deviation (SD), and numeric variables with no normal distribution were described as median and interquartile intervals (IQR). Categorical variables were described by absolute and relative frequencies.

The interobserver agreement was calculated using Cohen's Kappa statistics (linear weights for categorical variables and quadratic weights for ordinal variable). It was defined as: excellent (k ≥ 0.81), good (k = 0.61 – 0.80), moderate (k = 0.41 – 0.60), fair (k = 0.21 – 0.40) and poor (k ≤ 20).

To study the association between the explicative measurements and the biopsy results we used binominal logistic models and the discrimination was verified with area under the curve (AUC) receiver operating characteristic (ROC). Multiple model was acquired using stepwise process in both direction starting with a null model to a saturated model.

Analyses were performed using the software R 3.1.3 (R Core Team, 2015). The level for statistical significance was set at 5%.

## RESULTS

A total of 122 patients were enrolled in our study. Of the 60 positive biopsy results, 9 (15%) had Gleason score 3 + 3 and 51 (85%) had Gleason score ≥ 3 + 4. Median time between MRI and biopsy was 21 days. A median of 3 additional samples was obtained in each suspicious lesion on MRI.

Among all variables independently analyzed, ADC value was the best predictor of positivity for CS-prostate cancer on biopsy, with mean value of 1.08 mm^2^ / sec (SD 0.20) on negative biopsies and 0.81 mm2 / sec (SD 0.22) on positive biopsies for reader 1, and 1.09 mm^2^ / sec (SD 0.24) on negative biopsies and 0.84 mm^2^ / sec (SD 0.22) on positive biopsies for reader 2 (p < 0.001 in both analysis). AUC was 0.82 and 0.80 for reader 1 and 2, respectively (Table-1).

**Table 1:** Numeric variables included in the study.

	VARIABLES	NEGATIVE	POSITIVE	AUC (95%CI)	P VALUE
Reader 1	Size (mm)	7.00 [5.00; 11.00]	9.50 [7.75; 12.00]	0.64 (0.54-0.74)	0.048
	ADC-Value	1.08 (0.20)	0.81 (0.22)	0.82 (0.73-0.91)	<0.001
	Likert score	3.00 [2.00; 3.00]	4.00 [3.00; 5.00]	0.78 (0.70-0.85)	<0.001
	PIRADS score	3.00 [2.00; 3.00]	4.00 [3.00; 4.00]	0.77 (0.70-0.85)	<0.001
Reader 2	Size (mm)	10.00 [6.00; 14.00]	14.00 [9.75; 16.00]	0.65 (0.55-0.75)	0.010
	ADC-Value	1.09 (0.24)	0.84 (0.22)	0.80 (0.70-0.89)	<0.001
	Likert score	4.00 [4.00; 5.00]	5.00 [4.00; 5.00]	0.59 (0.50-0.68)	0.054
	PIRADS score	4.00 [4.00; 4.00]	4.00 [4.00; 5.00]	0.62 (0.54-0.71)	0.010

**AUC** = Area under the ROC Curve; **95%CI** = 95% confidence intervals; **OR** = estimated odds ratio

**Figure 1:**
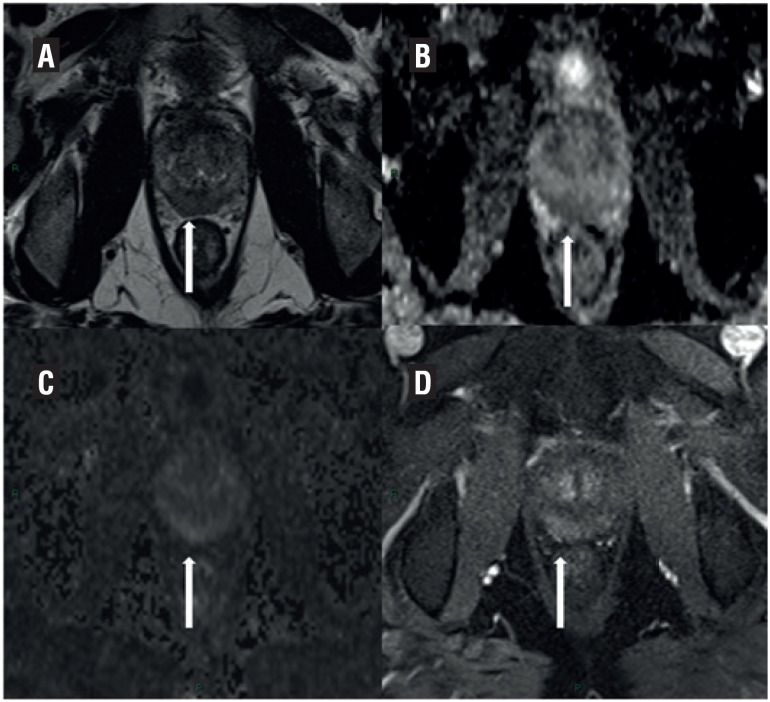
A nodule in the posterior right midgland of the peripheral zone (A). Reader 1 described as mild restriction diffusion on ADO map and diffusion (B and C) and no hyperenhancement (D), and final Likert and PIRADS score 3. Reader 2 described as moderate diffusion and hyperenhancement, and final Likert and PIRADS scores 5. Biopsy was negative for neoplasia.

For categorical variables (morphology, contours, signal in T2W, subjective intensity of diffusion restriction, hyper-enhancement, contact with surgical and prostatic capsule, and retraction of prostatic contour), the best AUC for reader 1 (Table-2) was subjective intensity of diffusion restriction (AUC of 0.74) and for reader 2 (Table-3) was hyper-enhancement (AUC of 0.65), all inferior comparing to the value of ADC map. Table-4 shows sensitivities, specificities, accuracies, positive and negative predictive for tumor, in presence of objective measurement of ADC value, being the most important variable that correlates with biopsy (Table-5) (Figure-1).

**Table 2:** Categoric variable for reader 1 in absolute number (%).

VARIABLES	CLASS	NEGATIVE (n=62)	POSITIVE (n=60)	AUC (95%CI)	P VALUE
Morphology	1-linear / v-shaped	11 (17.7)	3 (5.0)	0.62 (0.54-0.71)	
	2-oval	13 (21.0)	23 (38.3)		0.011
	3-nodular	38 (61.3)	34 (56.7)		0.086
Contours	1-well defined	28 (45.2)	17 (28.3)	0.59 (0.50-0.68)	
	2-partially defined	26 (41.9)	32 (53.3)		0.081
	3-undefined	8 (12.9)	11 (18.3)		0.142
Signal in T2	1-hypo	43 (69.4)	26 (43.3)	0.63 (0.54-0.72)	
	2-marked hypo	19 (30.6)	34 (56.7)		0.004
Signal in T2	1-homogeneous	18 (29.0)	36 (60.0)	0.65 (0.57-0.74)	
	2-heterogeneous	44 (71.0)	24 (40.0)		0.001
	1-mild	39 (62.9)	12 (20.0)	0.74 (0.66-0.83)	
ADC-intensity of diffusion restriction	2-moderate	17 (27.4)	26 (43.3)		<0.001
	3-marked	6 (9.7)	22 (36.7)		<0.001
Hyper-enhancement	0-absent	8 (15.1)	7 (12.7)	0.55 (0.44-0.65)	
	1-mild	19 (35.8)	17 (30.9)		0.971
	2-moderate	15 (28.3)	15 (27.3)		0.833
	3-marked	11 (20.8)	16 (29.1)		0.434
Contact with surgical	0-no	21 (33.9)	18 (30.0)	0.52 (0.44-0.60)	
capsule	1-yes	41 (66.1)	42 (70.0)		0.647
Contact with prostatic	0-no	23 (37.1)	10 (16.7)	0.60 (0.53-0.68)	
contours	1-yes	39 (62.9)	50 (83.3)		0.013
Retraction of prostatic	0-no	58 (96.7)	52 (94.5)	0.51 (0.47-0.55)	
contours	1-yes	2 (3.3)	3 (5.5)		0.581

**AUC** = Area under the ROC Curve; **95%CI** = 95% confidence intervals; **OR** = estimated odds ratio

**Table 3:** Categoric variable for reader 2 in absolute number (%).

VARIABLES	CLASS	NEGATIVE (n=62)	POSITIVE (n=60)	AUC (95%CI)	P VALUE
Morphology	1-linear / v-shaped	10 (16.1)	9 (15.0)	0.54 (0.45-0.63)	
	2-oval	19 (30.6)	23 (38.3)		0.593
	3-nodular	33 (53.2)	28 (46.7)		0.911
Contours	1-well defined	30 (48.4)	16 (26.7)	0.62 (0.52-0.71)	
	2-partially defined	25 (40.3)	36 (60.0)		0.014
	3-undefined	7 (11.3)	8 (13.3)		0.206
Signal in T2	1-hypo	43 (69.4)	35 (58.3)	0.56 (0.47-0.64)	
	2-marked hypo	19 (30.6)	25 (41.7)		0.206
Signal in T2	1-homogeneous	42 (67.7)	39 (65.0)	0.51 (0.43-0.60)	
	2-heterogeneous	20 (32.3)	21 (35.0)		0.749
	1-mild	18 (29.0)	6 (10.0)	0.64 (0.55-0.73)	
ADC-intensity of diffusion restriction	2-moderate	23 (37.1)	20 (33.3)		0.088
	3-marked	21 (33.9)	34 (56.7)		0.004
Hyper-enhancement	0-absent	9 (17.0)	3 (5.4)	0.65 (0.55-0.75)	
	1-mild	7 (13.2)	10 (17.9)		0.079
	2-moderate	12 (22.6)	24 (42.9)		0.018
	3-marked	25 (47.2)	19 (33.9)		0.261
Contact with surgical	0-no	20 (32.3)	20 (33.3)	0.51 (0.42-0.59)	
capsule	1-yes	42 (67.7)	40 (66.7)		0.899
Contact with prostatic	0-no	21 (33.9)	7 (11.7)	0.61 (0.54-0.68)	
contours	1-yes	41 (66.1)	53 (88.3)		0.005
Retraction of prostatic	0-no	51 (92.7)	52 (94.5)	0.51 (0.46-0.56)	
contours	1-yes	4 (7.3)	3 (5.5)		0.697

**AUC** = Area under the ROC Curve; **95%CI** = 95% confidence intervals; **OR** = estimated odds ratio

**Table 4:** Diagnostic measurements.

VARIABLE	POSITIVE	SENSITIVITY	SPECIFICITY	ACCURACY	PPV	NPV
**Reader 1**						
Size (mm)	≥ 7.5	0.75	0.53	0.64	0.61	0.69
Morphology	oval or nodular	0.95	0.18	0.56	0.53	0.79
Contour	Partially ill-defined or ill-defined	0.72	0.45	0.58	0.56	0.62
Signal in T2	marked hypo	0.57	0.69	0.63	0.64	0.62
Signal in T2	heterogeneous	0.40	0.29	0.34	0.35	0.33
ADC - subjective	moderate or marked	0.80	0.63	0.71	0.68	0.76
ADC - value	≥ 1010.5	0.16	0.29	0.23	0.17	0.27
Early enhancement	marked	0.29	0.79	0.54	0.59	0.52
Contact with prostatic contour	yes	0.83	0.37	0.60	0.56	0.70
Likert	4 or 5	0.55	0.85	0.70	0.79	0.66
PI-RADS	4 or 5	0.55	0.85	0.70	0.79	0.66
**Reader 2**						
Size (mm)	≥ 7.5	0.90	0.35	0.62	0.57	0.79
Morphology	Nodular	0.47	0.47	0.47	0.46	0.48
Contour	Partially ill-defined or ill-defined	0.73	0.48	0.61	0.58	0.65
Signal in T2	marked hypo	0.42	0.69	0.56	0.57	0.55
Signal in T2	heterogeneous	0.35	0.68	0.52	0.51	0.52
ADC - subjective	marked	0.57	0.66	0.61	0.62	0.61
ADC - value	≥ 910	0.30	0.12	0.20	0.22	0.17
Early enhancement	mild, moderate or marked	0.95	0.17	0.57	0.55	0.75
Contact with prostatic contour	yes	0.88	0.34	0.61	0.56	0.75
Likert	5	0.60	0.58	0.59	0.58	0.60
PI-RADS	5	0.35	0.89	0.62	0.75	0.59

**PPV** = positive predictive value; **NPV** = negative predictive value

**Table 5:** Multiple models to identify positive biopsy.

	COEFFICIENTS	ESTIMATE	OR (95%CI)	P VALUE
Reader 1	Intercept	-4.09		<0.001
AUC	Contact with prostatic contour (present)	1.42	4.14 (1.50-11.43)	0.006
0.82 (0.74-0.89)	ADC - Intensity moderate or marked	2.19	8.93 (3.43-23.29)	<0.001
	Contours (partially defined or ill-defined)	1.09	2.98 (1.17-7.58)	0.022
	Morphology (oval or round)	1.14	3.13 (0.65-14.96)	0.153
Reader 2	Intercept	4.77		0.001
AUC	ADC - value	-0.01	0.995 (0.993-0.997)	<0.001
0.82 (0.74-0.91)	Contact with surgical capsule (present)	-0.95	0.38 (0.13-1.13)	0.083
	Contours (partially defined or ill-defined)	0.89	2.42 (0.88-6.65)	0.086

Boxplots graphics shows that subjective impression to categorize the intensity of restriction (mild, moderate and marked) had direct strong negative correlation with ADC values, with coefficients of −0.83 (confidence interval of 95%: −0.90 to −0.74) for reader 1, and −0.64 values of both readers in the features that had clinically significant differences on independent analysis.

Interobserver agreement ranged from 0.13 to 0.75, poor in most measurements, and good or excellent (kappa > 0.6) only in lesion size and ADC values (both numeric variables).

### Multiple models

**Figure 2:**
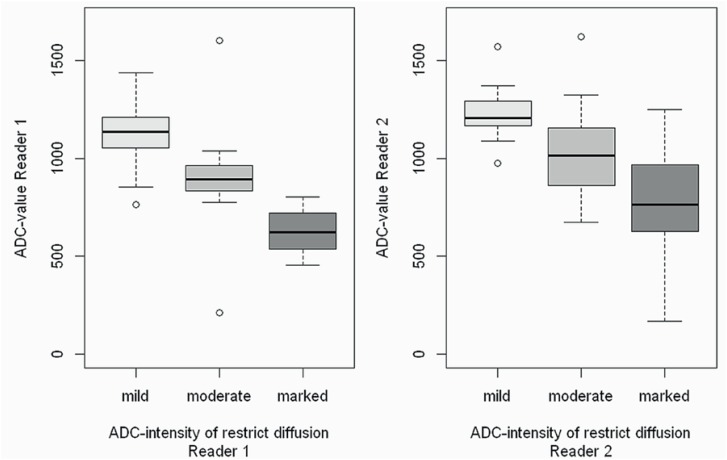
Graphics presented in boxplot showing the correlation of subjective ADC map grade and the objective ADC map value.

Multiple models analyses were obtained independently for readers 1 and 2. For reader 1, variables that remained significantly associated with tumor on biopsy, in presence of ADC value, were: contact with prostatic contour, subjective restriction diffusion on ADC map (moderate and marked), and lesion contours partially defined or ill defined. For reader 2, none of the variables were significant associated with positive biopsy (confidence interval of 95%: −0.75 to −0.51 for reader 2). However, this association was stronger for reader 1 than for reader 2, who had high overlap on ADC values measurements for subjective categorization of moderate and marked diffusion restriction (Figure-2).

## DISCUSSION

Our study showed that objective value of lesion's diffusion restriction, measured on ADC map, is the best predictive variable for prostate cancer. For the more experienced reader, the subjective value of ADC (moderate and marked) also had significant correlation with positive biopsies. However, for less experienced reader, the subjective variable had high overlap and showed no correlation with positive biopsies on multivariate analysis.

Prostate MRI is a method routinely performed to detect prostate cancer prior to first biopsy or after a negative biopsy (1, 2, 7). A structured prostate report is a major contribution for prostate MRI examination, and represents the initial steps to do a widely available method to detect prostate cancer. However, it is a challenging method with low to moderate rates of interobserver agreement even with standardized methodology (14, 15).

It is already known that there is a correlation between the grade of diffusion restriction in the prostate and the presence of cancer (1620). Our study shows that the subjective graduation of restricted diffusion can be misclassified for less experience readers, what could imply the final categorization of a prostatic lesion on MRI.

The variability in the subjective analysis probably explains the low rates of interobserver agreement. Median ADC values in category moderate and marked was 0.89 mm2 / sec (IQR 0.88 – 0.96) and 0.62 mm2 / sec (IQR 0.54 – 0.72), respectively, for reader 1, and 1.02 mm2 / sec (IQR 0.86 – 1.15) and 0.76 mm2 / sec (IQR 0.63 – 0.96), respectively, for reader 2. Note that for reader 2 there is an interpolation for moderate and marked restriction diffusion ranging from value from 0.86 mm^2^ / sec to 0.96 mm^2^ / sec (Figure-2). We believe that an objective cutoff for ADC map value, included in routine practice, would increase interobserver agreement to categorize lesions with mild, moderate and marked restriction diffusion, contributing to the standardize methodology become more reproducible. To the best of our knowledge, there is no data suggesting using objective ADC map values to categorize the grade of restriction diffusion.

There are some factors described that can affect the ADC measurements, such as b values, respiration conditions, field strength, vendor and other technical parameters (21). On the other hand, Sadinski et al. demonstrated, comparing ADC maps between two consecutive scans of same patient, that the reproducibility of ADC measurements in prostate is reasonable, suggesting that quantitative values obtained in DWI-MRI of prostate cancer are reproducible (22). Our study found similar values on ADC map for positive and negative prostate cancer biopsies (0.81 mm2 / sec and 1.08 mm2 / sec for reader 1, and 0.84 mm2 / sec and 1.09 mm2 / sec for reader 2, respectively), compared to literature, that ranges from 0.74 mm2 / sec (SD 0.15) to 0.80 mm^2^ / sec (SD 0.25) for positive prostate cancer biopsies and 1.35 mm^2^ / sec (SD 0.31) to 1.48 mm2 / sec (SD 0.29) for negative biopsies (18, 19), corroborating that ADC map value can be reproducible. Also, it has been already shown that using a parameter to normalize the ADC measurement (as the normal parenchyma or muscle), the ADC value can be reproducible among different scanners (21).

All others variables included in this study (size, Likert and PI-RADS classifications, morphology, contours, signal in T2W, contact with surgical capsule and prostatic contours, prostatic contours retraction and hyper-enhancement) were less or no important comparing to the intensity of diffusion restriction on ADC map.

Rosenkrantz et al. (23) recently proposed adjustments in PI-RADS version 2: in transition zone upgrade score 3 to 4 based on diffusion restriction score of 4 or modified dynamic contrast enhanced positive when incorporating new criteria, and in transition or peripheral zones upgrade score 4 to 5 based on size of 10-14 mm. Our study also showed that intensity of diffusion restriction is the most important variable that correlates with cancer, even in transition zone, and indeed our present observation provides a solid background to upgrade the PI-RADS score 3 to 4 when there is moderate diffusion restriction instead of considering only marked diffusion restriction. Also, size of the lesions had a median value of 9.5 mm and 14 mm in positive biopsies for readers 1 and 2, respectively, and did not correlated with positive biopsy in multiple models analyses. So, we agree that a 10 mm threshold would be a better cutoff to differentiate scores 4 and 5.

Our study has some limitations: first, we had only two readers with different levels of experience. Second, the readers evaluated only the previously specified lesions and not the whole gland; we chose this methodology because the reference standard used was the fusion biopsy (using US and RM images) and we wanted to be sure that the analyzed lesion was the biopsied one. Also, we aimed to compare the interobserver agreement and assess the variables for the same abnormality. Third, we did not analyze lesions in the peripheral and transition zones in subgroups. Finally, we did not measure the relative ADC map value.

## CONCLUSIONS

Diffusion restriction with lower ADC-values is the best parameter to predict cancer on multiparametric MRI prior to biopsy. Efforts to establish an ADC cutoff value would improve cancer detection, especially for less experience readers. Also, developing ADC as a quantitative imaging marker would allow better detection of prostate cancer by reducing inter-radiologist subjectivity, improving MRI results and therefore avoiding unnecessary biopsy, reducing overdiagnosis and overtreatment of prostate cancer.

### Clinical Relevance

Prostate MRI is evolving for the diagnosis of cancer. There is still debate in literature to prove the best method for diagnosis, but none of them include ADC objective values to make the differentiation between benign and malignant lesions.
